# Supplementary material for: Content-rich biological network constructed by mining PubMed abstracts
Source: BMC Bioinformatics. 2004 Oct 8;5:147. doi: 10.1186/1471-2105-5-147 (PMC528731; doi:10.1186/1471-2105-5-147)
Supplement: Additional File 2 — The original results of the above study (non-essential files are deleted to keep the file size under the limit set by BMC bioinformatics). [file 1471-2105-5-147-S2.bz2 › chilibotAdditionalFile2/dip05/715ID11744735E18052/html/CCME_CCMF.html]

 


 **CCME** and **CCMF** 
  
Found 5 abstracts in PubMed, retrieved 05.  
 

 What does Google say? 
 PDF only 
| .edu only 

---

**Interactive relationship** (e.g. stimulation, inhibition, etc)

**Neutral relationship**- Co immunoprecipitation experiments revealed that  **CcmF**  interacts directly with the heme donor  **CcmE**  and with CcmH but not with apocytochrome c.  Ref: 11744735 J Biol Chem, 2002
- **CcmF**  forms a complex with the heme chaperone  **CcmE**  and CcmH but not with apocytochrome c.  Ref: 11744735 J Biol Chem, 2002
- CcmC is required for binding of haem to  **CcmE** , and  **CcmF**  for releasing it from  **CcmE**  and transferring it onto the apocytochrome.  Ref: 11004446 Biochim Biophys Acta, 2000
- Both haem binding to and release from  **CcmE**  involve integral membrane proteins, CcmC and  **CcmF**  respectively, which carry a conserved tryptophan rich motif in a periplasmic domain.  Ref: 12196152 Biochem Soc Trans, 2002
- The haem dependent peroxidase activity of  **CcmE**  is clearly visible not only in a  **ccmF**  mutant, but also in ccmG and ccmH mutants, implying that  **CcmE**  functions either before or in the same step as  **CcmF** , CcmG and CcmH in cytochrome c maturation.  Ref: 9770295 FEMS Microbiol Lett, 1998
- **CcmE**  is a periplasmic heme chaperone that binds heme covalently and transfers it onto apocytochrome c in the presence of  **CcmF** , CcmG, and CcmH.  Ref: 10339610 Proc Natl Acad Sci U S A, 1999

**Non-interactive relationship** (e.g. studied together, co-existance, homology, etc.)

- We previously reported that a 17.5 kDa haem binding polypeptide accumulates in Escherichia coli K 12 mutants defective in an essential gene for cytochrome c assembly,  **ccmF** , and speculated that this polypeptide is either  **CcmE**  or CcmG.  Ref: 9770295 FEMS Microbiol Lett, 1998
- At least three of them, CcmC,  **CcmE**  and  **CcmF** , are thought to interact directly with haem.  Ref: 11004446 Biochim Biophys Acta, 2000
- Our data suggest that haem transferred across the cytoplasmic membrane by the CcmABCD complex becomes associated with  **CcmE** , possibly by a labile covalent bond, before it is transferred to the cytochrome c apoproteins by the periplasmic haem lyase encoded by  **ccmF**  and ccmH.  Ref: 9770295 FEMS Microbiol Lett, 1998
